# Designing and assessing a data literacy internship program for graduate health sciences students

**DOI:** 10.5195/jmla.2022.1498

**Published:** 2022-10-01

**Authors:** Bethany Sheriese McGowan, Abigail Ekeigwe, Kari Clase

**Affiliations:** 1 bmcgowa@purdue.edu, Associate Professor, Libraries and School of Information Studies, Purdue University, West Lafayette, IN.; 2 aekeigwe@purdue.edu, Research Assistant, Agricultural and Biological Engineering, Purdue University, West Lafayette, IN; 3 klclase@purdue.edu, Professor of Agricultural and Biological Engineering, Purdue University, West Lafayette, IN

**Keywords:** Data literacy, systematic review, internship, training, qualitative, data analysis, NVivo

## Abstract

This case study presents the results of a data internship and workshop series on data analysis in qualitative biomedical systematic reviews. In a newly developed librarian-led internship program, an intern was trained on data literacy concepts and data analysis tools and, in turn, helped recruit and train other graduate health sciences students. Due to COVID-19 restrictions, a flipped classroom model was applied to develop a completely virtual learning experience for both the intern and workshop attendees. Both the data intern and workshop participants reported improved confidence in data literacy competence at the end of the project. Assessment results suggest that while the workshop series improved participants' data literacy skills, participants might still benefit from additional data literacy instruction. This case also presents a model for student-led instruction that could be particularly useful for informing professional development opportunities for library interns, fellows, and student assistants.

## BACKGROUND

This project, the creation of a National Network of Libraries of Medicine-funded data literacy internship, seeks to build capacity in the library and information science community by supporting data-driven research. The internship has two overall objectives: 1. to train a data intern to develop proficiency in issues related to data analysis for qualitative systematic reviews, and 2. to support the intern in developing and leading peer training on data analysis for qualitative systematic reviews.

In the case presented in this study, the intern was a graduate student studying biotechnology innovation and regulatory science. The ability to synthesize evidence using critical methods to identify, define, and assess research on a clearly presented topic has become a critical skill in biotechnology innovation, especially with the introduction of the European Union (EU) Medical Devices Regulation (MDR). The EU MDR requires manufacturers of biomedical products for human use to submit a Clinical Evaluation Report (CER), which includes expert appraisal and analysis of clinical evidence sources [1]. In addition to their use informing the design of new medical devices, qualitative and systematic approaches are used to inform clinical trial design [2], medical device development [3], and policymaking [4]. Studies have highlighted the need for SR-related training in biotechnology innovation education [6]. The specific need for data analysis training as related to qualitative systematic review methods in the Biotechnology Innovation and Regulatory Science Center (BIRS) program at Purdue University stimulated the formation of this internship program.

This project follows the launch of a graduate-level systematic review course by the Purdue University Libraries and School of Information Studies (the Libraries) in 2019 [7]. The data intern completed ILS 595: Introduction to Systematic Review for the Health Sciences in Spring 2019. ILS 595 is a one-credit Libraries course that teaches biomedical systematic review methodology. Over the subsequent 18-months, the intern worked with a librarian–one of the ILS 595 course instructors–and led a research team conducting a qualitative systematic review that described the competency requirements of regulatory scientists in sub-Saharan Africa [8]. Their work highlighted a need for qualitative data analysis and systematic review training for graduate students in the BIRS program, the intern's home program. The collaboration between the Libraries and BIRS was strengthened, and NNLM funding was secured to establish a data internship program and launch a training opportunity.

In the first half of the data internship, the intern received advanced training on research methods and research ethics related to systematic reviews and meta-analyses. Both topics had been covered to a basic proficiency level in ILS 595; however, the internship focused specifically on qualitative systematic review methodology, as opposed to the quantitative focus of ILS 595. According to the Cochrane Qualitative and Implementation Methods Group Guidance Series, qualitative review questions differ from quantitative review questions in that they ask 'how' and 'why'; are exploratory; aim to identify what is known from multiple perspectives; reveal different factors, dimensions, and explanations; and may initially be broad to map what is known before formulating or refining [9]. The data intern also received training on topics related to open science and FAIR data use and was introduced to student-centered learning pedagogy. The plan of study used to train the data intern is available in the Supplemental Material.

In the second half of the data internship, the intern developed and led a 3-part workshop series related to qualitative data analysis for systematic reviews and meta-analyses. The training included an introduction to text analysis using NVivo 12 software. Though the training was marketed campus and community-wide, its primary target audience was the graduate students in the BIRS program, which includes both United States-based and African-based cohorts. The BIRS program is as housed within the Department of Agricultural and Biological Engineering (ABE) at Purdue University. Programs within ABE equip students to tackle complex problems that lie at the intersection of science and engineering, such applying biology and technology to address problems in health and pharmaceutics. In recent years, the Libraries has grown its capabilities for supplementing on-campus instruction, and many students in the BIRS program benefit from both the online tutorials, electronic databases, and accessibility of expert guidance from librarians on their graduate research projects. Goals for the workshop series were aligned with some of the competencies for the BIRS program, including effective communication and the development of research and critical thinking skills to implement evidence-based solutions.

BIRS graduate students in the most recent cohorts had been admitted in Fall 2020 and when the workshop series launched in Spring 2021 were still becoming familiar with Libraries resources and with the relevance of systematic reviews. In addition to boosting workshop participants' knowledge of research methods related to data analysis in qualitative systematic reviews, the internship and workshop series helped the Libraries build capacity and market its databases, datasets, institutional repository, and instructional services–especially its newly launched graduate courses.

## CASE PRESENTATION

This study was approved by the Purdue University Institutional Review Board as IRB-2021-402 titled 'Building the Data Literacy Competencies of Graduate Health Sciences Students'.

### Data Internship

#### Internship Structure

The librarian met with and trained the data intern for one hour, once a week, over twelve weeks. The training followed a flipped learning model. Before each meeting the librarian shared training materials with the data intern, then meeting times were used to answer outstanding questions and clarify crucial concepts. NNLM-funding was used to compensate the intern as a Graduate Assistant for the 12-week internship period. Due to COVID-19 restrictions, the intern's training was conducted virtually using Zoom.

#### Internship pre-assessment and learning outcomes development

The internship's learning outcomes were based on a learner-centered, competency-based learning model tailored to meet the specific needs of the intern. The librarian distributed a pre-assessment in which the data intern self-assessed their data literacy competence, knowledge of research methods, understanding of research ethics and rigor, perceived importance of data literacy competencies, and familiarity with open science practices. Data Literacy's *Self-Assessment: 17 Key Traits of Data Literacy,* a validated tool for assessing data literacy, was used for both the pre- and post-assessments [10]. Using the pre-assessment, the data intern scored each of the seventeen traits according to how proficient they felt on a scale of zero to four, and how important they felt each trait was to their professional success, also on a scale of zero to four. According to the assessment calculation, the Importance Score minus the Proficiency Score can be used to determine a Learning Priority Score for each trait. The learning priority scores could range from -4 (very low learning priority) to +4 (very high learning priority). Pre-assessment results were used to identify the intern's learning priorities and to inform the development of the internship's learning outcomes, content, and structure.

The internship's learning objectives were that after completion of the internship, the intern will be able to:

1. Apply FAIR data principles to manage data;
2. Use Tableau to create data visualizations; and
3. Co-develop and co-lead a data literacy instructional series focused on qualitative data analysis for biomedical systematic reviews and evidence syntheses.

#### Pre-and Post-Assessment Analysis

**Figure 1:**
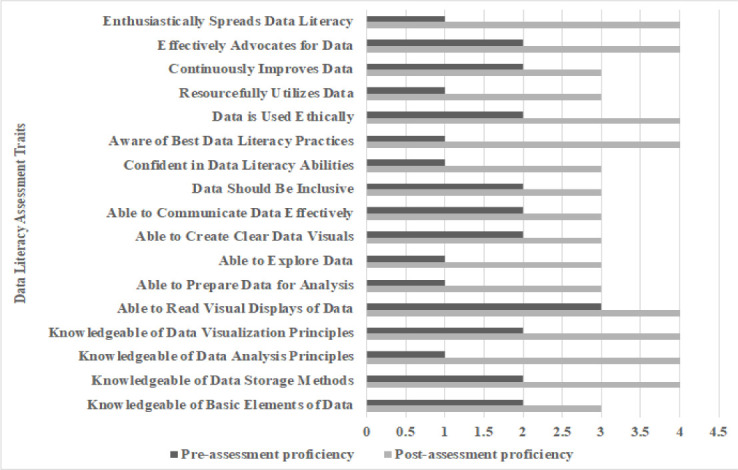
Data intern's self-reported pre- and post-proficiency on 17 Key Traits of Data Literacy.

The results from a comparison of the data intern's pre-and post-self-assessments proficiency on the Data Literacy's *Self-Assessment: 17 Key Traits of Data Literacy* (Figure 1) suggest that the data internship met its learning objectives. At the end of the internship's first six weeks, the intern self-reported improved proficiency on all seventeen traits. Further, the intern demonstrated their proficiency by organizing and teaching a three-part workshop series that trained other students on data analysis for qualitative biomedical systematic review.

Reflecting on their experience in the internship, the data intern indicated that the one-on-one guided training and the flipped class approach broke the initial intimidation about data-driven research and enabled their learning. However, they suggested that the internship period could be increased from one semester to one academic year to allow for more hands-on practice with tools. She reported:

**“At the start of the data internship, being naïve to data analysis, I was anxious about achieving the learning outcomes of the program. However, reading and digesting the internship's materials ahead of the learning sessions and the one-on-one interaction with my instructor allayed my initial trepidation and enabled my learning.”**

### Structure of the Workshop Series

The intern developed a 3-part workshop series based on a flipped classroom learning model. Instruction for the series followed a flipped classroom learning approach in which learners worked individually and asynchronously to learn the essential instructional content before coming to class, then synchronous meetings were used for collaborative, problem-solving activities that assisted them in internalizing and constructing their knowledge and developing new competencies. This student-centered model, based on the constructivist theory of learning, is effective in different disciplines and is especially useful for virtual instruction [11, 12].

The workshop series included three asynchronous training videos and three synchronous learning sessions. The data intern, the librarian, and the director of the BIRS program (a faculty member in the College of Agriculture/Agriculture and Biological Engineering) co-developed the three videos that taught the essential content of the workshop series. The first video focused on knowledge of research methods, emphasizing methods related to conducting qualitative systematic reviews. The second video focused on the use of NVivo to support data management and qualitative data analysis. And the third video introduced research ethics, FAIR data principles, and open access practices. The video recordings were uploaded to the BIRS Center YouTube page and links to the videos were shared with registered participants via email. Workshop registrants were instructed to review the videos before each synchronous session. The synchronous sessions were used for hands-on practice with NVivo, collaborative activities on the systematic review process, and question and answer sessions.

### Workshop Logistics

Email was used for all correspondences with participants and the pre-assessment was shared with participants via a Qualtrics survey link a week before sharing the asynchronous videos. The asynchronous videos were shared a week before each synchronous session. All synchronous sessions were hosted virtually on Zoom. Participants were encouraged to obtain and download the NVivo software before the synchronous sessions. Purdue University provides NVivo licenses to graduate students at no charge. Workshop instructors used a mini-project worksheet to guide participants as they practiced functions with NVivo. This worksheet is available in the Supplemental Material. Results of student performance on the worksheet are discussed in a later section. At the end of the workshop series, the instructors emailed participants a link to the post-assessment via a Qualtrics survey. In line with the FAIR principles, all workshop materials are published in the open-access Purdue University Research Repository (https://purr.purdue.edu/publications/3875/1) [13].

### Workshop Participants Demographics

The workshop series was primarily advertised to graduate students in health sciences disciplines by emailing students who had previously attended Libraries' systematic review-related training. Fifty students registered for the workshop. Most students were from the BIRS program (n=46), with others from Agricultural and Biological Engineering (n=1), Biomedical Engineering (n=1), and Speech, Language, and Hearing Sciences (n=2).

### Determining Learning Gains of Workshop articipants

Workshop attendees' pre- and post-self-assessment results, and performance results on the end-of-training mini-projects, were used to measure learning gains. The pre-and post-assessments both comprised the same questions. The assessments consisted of twelve traits. Workshop attendees scored each of the twelve traits according to how proficient they felt on a scale of zero to four, and how important they felt each trait was to their professional success, also on a scale of zero to four. The pre/post assessment is available in the supplemental material.

**Figure 2:**
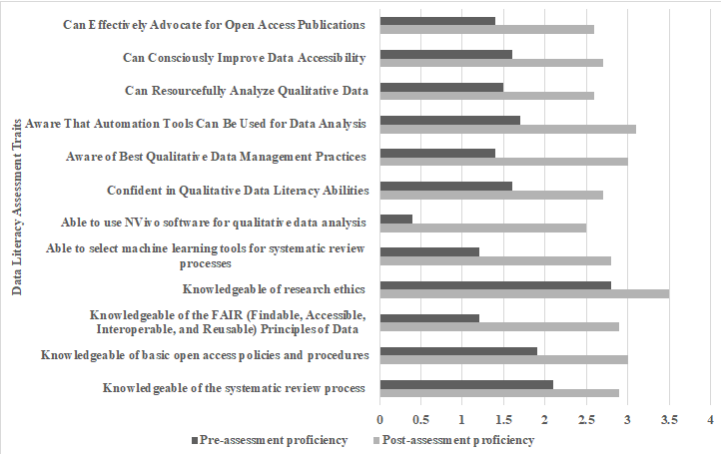
Workshop participants' self-reported pre-and post-proficiency on data literacy

We used Excel to conduct a comparative analysis of workshop attendees' pre-and post-self-assessed data literacy proficiency. The aggregate self-reported proficiency of workshop participants increased between the pre-and post-assessments for all data literacy items in the assessment instrument (Figure 2). The increase in proficiency for all traits indicates net participants' learning from the workshop series. However, there is still room for growth opportunities since self-estimated proficiency for all assessment items are not yet at the maximum score.

Generally, participants indicated increased awareness of the systematic review process, the FAIR and open-access principles, and the use of NVivo in qualitative systematic reviews. For example, in describing their understanding of systematic review methods one workshop participant noted: “I now understand the difference between a routine literature review and the systematic review process. The rigor of systematic reviews is important in biomedical sciences, especially in the performance evaluation of medical devices. Thus, I will apply my learnings from this course to my professional practice and graduate studies.”

Participants expressed interest in learning more about the systematic review process since they believed that the workshop series was too brief to allow for mastery of the process. And, discussions related to open science highlighted the need for financial support, with one participant noting: “I appreciate the open-access principles; however, institutions and libraries can promote open-access by assisting graduate students to pay publishing fees.”

Eleven participants (22%) opted to submit their workshop mini project for grading. Generally, the mini project results indicated that participants learned the systematic review process. However, they had challenges developing search strategies and developing proficiency using NVivo software. Participants were invited to register for the Purdue Libraries' systematic review courses for more robust instruction related to these competencies.

## DISCUSSION

The data internship model of training a data intern who in turn trains a larger group of peers is illustrative of the effectiveness of the train-the-trainer model in education [14, 15]. The data intern honed competencies in the FAIR data use and open-access principles, the use of Tableau to visualize data, and the systematic review process, while simultaneously improving their presentation skills and increasing awareness of the library's resources and courses. Workshop participants increased their awareness of the FAIR data and open-access principles, systematic review process, and the use of NVivo 12 software in qualitative systematic review.

The flipped classroom model (a student-centered instructional technique) implemented in this project was effective because it gave learners the autonomy to choose convenient times for learning [16]. We found that the model worked well for both the data internship and the workshop series, allowing participants to practice and implement core concepts on their own before the synchronous sessions. Further, the use of a pre-assessment that measured how important participant's felt individual data literacy traits were to their professional success allowed instructors to create highly relevant learning opportunities.

Findings from previous studies suggest that workshop training can help improve the competencies of participants to a certain level but further training might still be needed; for example, in professional development workshops for teachers [17]. We came to a similar conclusion with this case study. While the workshop series met its learning objectives, results from our assessment of attendees suggest that more data literacy instruction would be helpful, especially for complex tasks like building a robust search strategy and learning to use new software. A full course or having these competencies integrated throughout a program curriculum could be beneficial. As an immediate next step, the BIRS program incorporated the materials from the workshop as resources for graduate students in its research seminar course and the libraries plans to rely on the findings from this study to inform future collaboration with other graduate health sciences programs.

This workshop series introduced data analysis techniques, the use of NVivo 12 software, and an introduction to FAIR data principles. Learning workshops, such as the series presented in this case study, are designed to help participants receive practical training on a given subject [18]. The hands-on activities included are usually more effective when in traditional face-to-face settings. Thus, it is not surprising that participants in this virtual workshop series noted challenges in completing the hands-on activities during the virtual live synchronous sessions. Also, the virtual Zoom format made it difficult for instructors to help participants individually as they worked through the activities during the online synchronous session. To remediate this issue, all synchronous sessions were recorded and shared with participants to work through the process on their own. Participants were encouraged to email questions and received prompt guidance from the workshop instructors through emails and additional virtual practice sessions, when needed. Future action might include breaking participants into smaller virtual breakout groups where they work as a team, allowing for peer-to-peer support and allowing instructors to more easily drop in and offer guidance.

Another concern included a recognition of the importance of open science practices but a need for financial support for open access publishing. Future action could include emphasizing the ways the Purdue Libraries supports open access publishing, including an open access publishing support fund for gold OA journals; partnerships with select publishers to remove open access fees; and green open access options in the institutional repository.

Another challenge is sustainability of the internship program. An NNLM grant funded the program for one iteration and was wholly used to pay the intern's salary. Future action might include securing a larger grant to test the internship for several additional iterations and extending the internship experience to a year-long experience, as recommended by the intern in this case study. We might then use the resulting evidence to support the proposal for a permanent Libraries-funded internship position.

## CONCLUSION

The data internship and workshop training presented in this study support the Network of National Libraries of Medicine's (NNLM) goal to “build students' professional competencies in the field of data science and research data management” [19]. The data intern was trained on data literacy concepts and data analysis tools, and in turn, developed an approach to recruit and train other graduate students. This two-part intern training model could be particularly useful for informing professional development opportunities for interns, fellows, and student assistants. Based on their post-internship feedback, allowing the intern to design and lead the workshop series provided a valued sense of autonomy that is sometimes missing from internship and residency experiences. Having a student-led workshop series' marketing campaign added valuable insight into strategies for reaching and engaging students. Also, the flipped classroom model was especially useful to the virtual workshop setting and helped ensure that participants had access to all the materials before, during, and after the series. Both the data intern and participants reported improved confidence in data literacy competence at the end of the training. Assessment results suggest that while the workshop series improved participants' data literacy skills, participants could benefit from additional data literacy instruction. The Libraries offers or plans to offer a range of learning experiences to address these needs.

## Data Availability

The data literacy assessment tool; the workshop videos and PowerPoint slides; data tables, and results of the quantitative analysis on Tableau are available in the Purdue University Research Repository (20).
